# The Wound Dresser

**DOI:** 10.14797/mdcvj.1128

**Published:** 2022-06-03

**Authors:** James B. Young

**Affiliations:** 1 Executive Director of Academic Affairs, Cleveland Clinic, Professor of Medicine, Cleveland Clinic Lerner College of Medicine of Case Western Reserve University, Cleveland, Ohio, US

## 1

An old man bending I come among new faces,
Years looking backward resuming in answer to children,
Come tell us old man, as from young men and maidens that love me,
(Arous’d and angry, I’d thought to beat the alarum, and urge relentless war,
But soon my fingers fail’d me, my face droop’d and I resign’d myself,
To sit by the wounded and sooth them, or silently watch the dead;)
Years hence of these scenes, of these furious passions, then these chances,
Of unsurpass’d heroes, (was one side so brave? The other was equally brave;)
Now be witness again, paint the mightiest armies of earth,
Of those armies so rapid so wondrous what saw you to tell us?
What stays with you latest and deepest? of curious panics,
Of hard-fought engagements or sieges tremendous what deepest remains?

## 2

O maidens and young men I love and that love me,
What you ask of my days those the strangest and sudden your talking, recalls,
Soldier alert I arrive after a long march cover’d with sweat and dust,
In the nick of time I come, plunge into the fight, loudly shout in the rush of successful charge,
Enter the captur’d works—yet lo, like a swift running river they fade.
Pass and they are gone they fade—I dwell not on soldiers’ perils or soldiers’ joys,
(Both I remember well—many of the hardships, few the joys, yet I was content.)

But in silence, in dreams’ projections,
While the world of gain and appearance and mirth goes on,
So soon what is over forgotten, and waves wash the imprints off the sand,
With hinged knees returning I enter the doors, (while for you up there,
Whoever you are, follow without noise and be of strong heart.)
Bearing the bandages, water and sponge,
Straight and swift to my wounded I go,
Where they lie on the ground after the battle brought lit in,
Where their priceless blood reddens the grass, the ground,
Or to the rows of the hospital tent, or under the roof’d hospital.
To the long rows of cots up and down each side I return.
To each and all one after another I draw near, not one do I miss,
An attendant follows holding a tray, he carries a refuse pail,
Soon to be filled with clotted rags and blood, emptied, and fill’d again.

I onward go, I stop.
With hinged knees and steady hand to dress wounds,
I am firm with each, the pangs are sharp yet unavoidable,
One turns to me his appealing eyes—poor boy! I never knew you,
Yet I think I could not refuse this moment to die for you, if that would save you.

## 3

On, on I go, (open doors of time! Open hospital doors!)
The crushed head I dress, (poor crazed hand tear not the bandage away,)
The neck of the cavalry-man with the bullet through and through I examine.
Hard the breathing rattles, quite glazed already the eye, yet life struggles hard,
(Come sweet death! Be persuaded O beautiful death!
In mercy come quickly,)

From the stump of the arm, the amputated hand,
I undo the clotted lint, remove the slough, wash off the matter and blood,
Back on his pillow the soldier bends with curv’d neck and side falling head,
His eyes are closed, his face is pale, he dares not look at the bloody stump,
And had not yet look’d on it.

I dress a wound in the side, deep, deep,
But a day or two more, for see the frame all wasted and sinking,
And the yellow-blue countenance see.

I dress the perforated shoulder, the foot with the bullet-wound,
Cleanse the one with the gnawing and putrid gangrene, so sickening, so offensive,
While the attendant stands behind aside me holding the tray and pail.

I am faithful, I do not give out,
The fractured thigh, the knee, the wound in the abdomen,
These and more I dress with impassive hand, (yet deep in my breast a fire, a burning flame.)

## 4

Thus in silence, in dreams progression,
Returning, resuming, I thread my way through the hospitals,
The hurt and wounded I pacify with soothing hand,
I sit by the restless all the dark night, some are so young,
Some suffer so much, I recall the experience sweet and sad,
(Many a soldier’s kiss dwells on these bearded lips.)

Walt Whitman, 1819–1892
*Drum-Taps*, 1865
This poem is in the public domain.

## Conflict, compassion, and whitman’s nursing

Daily reports of senseless, brutal, and massive civil destruction with extraordinary human pain and suffering remind us, yet again, of another “almost” world war, this time in Ukraine. A sad fact is that war rages nonstop every day somewhere in our world. Some conflicts are ignored, but others attract intense focus because of real or perceived threats to our nation. All, however, are horrific testaments to persistent inhumanity that plagues mankind. Indeed, in late August 2021 the United States exited, but not necessarily ended, a 20-year war in Afghanistan, the longest in our history there.

**Figure:**
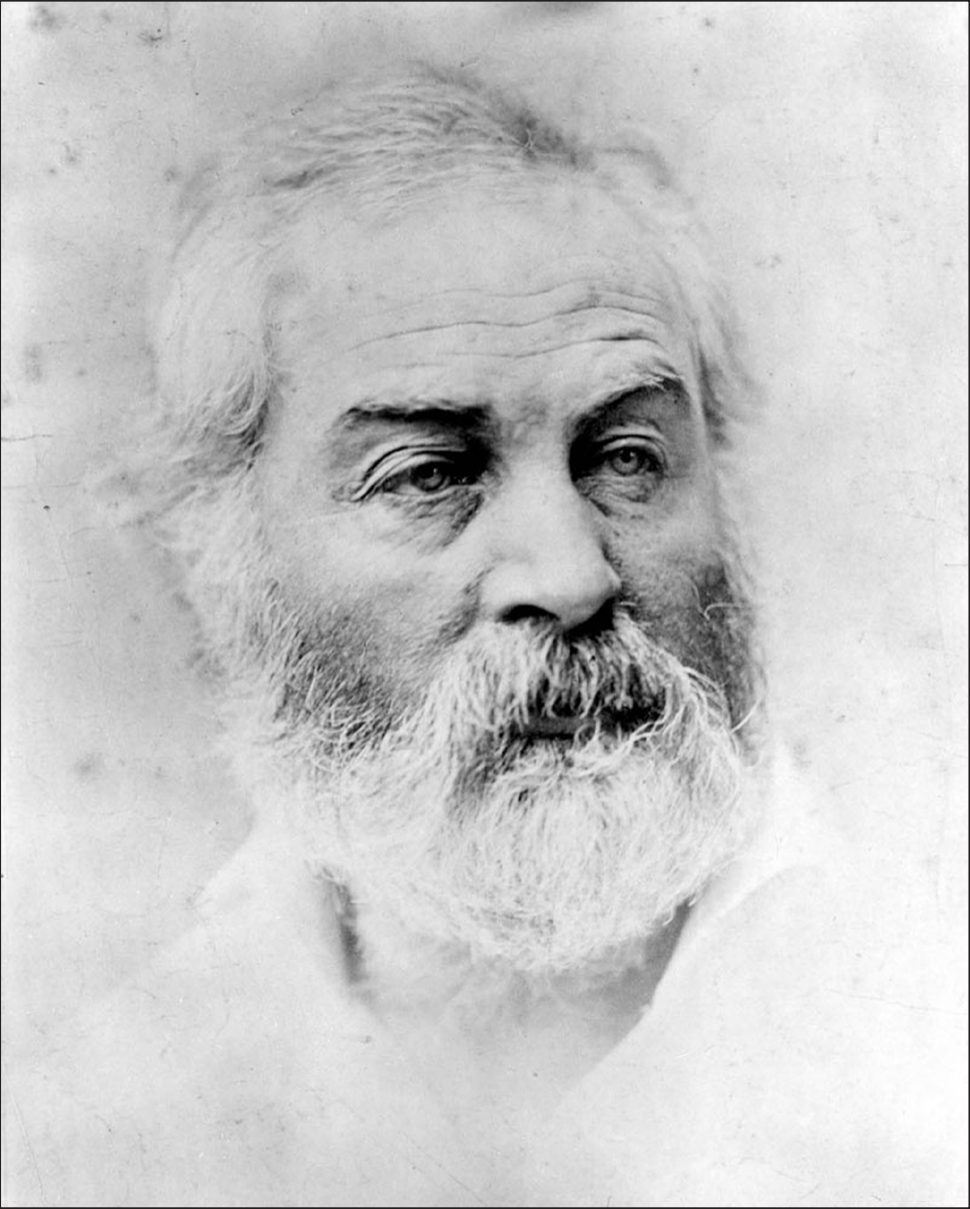
Walt Whitman by Alexander Gardner, ca. 1863-1864. Digital image was created by Clifton Waller Barrett Library of American Literature, Albert and Shirley Small Special Collections Library, University of Virginia. This image is in the public domain and may be reproduced without permission.

The American Civil War, one focus of Walt Whitman’s poetry, was the last war fought on our lands unless you count the 9/11 attacks. But injury, suffering, and death of our soldiers and citizens during conflict continues, nonetheless. Though sad and depressing, some argue that war is simply a burden of the human race and civilization. Art and literature, including poetry, poignantly capture the courage, heroism, folly, suffering, and compassion that accompany conflict. Remember Pablo Picasso’s painting on the tragedy of war, *Guernica* (1937), Robert Capa’s photograph of the *Falling Soldier* blown away while moving across a battlefield (1938), or Ernest Hemingway’s poignant novel, *For Whom the Bell Tolls* (1940)? Each presents a different art form focused on the Spanish Civil War to highlight the tragedy of war.

More than a few Walt Whitman poems join this important and heroic category of literature that compels us to share, at least intellectually, the agony of battle. We are forced to see the pain and waste of conflict that reaches back to prehistoric and ancient times; note the literature devoted to the Persian, Peloponnesian, and Trojan wars of Greece.

Whitman, who was born on Long Island in 1819 and moved to Brooklyn in his early years, had only a rudimentary and peripatetic education in local public schools. Like Benjamin Franklin, he became a printer, newsman, and journalist but also spent a good deal of his life as a teacher. In 1855, Whitman published the first edition of his major iconic work, *Leaves of Grass*, which subsequently went through nine editions with poems focused on nature, friendship, tragedy, democracy, and love. Over time, Whitman was applauded widely and was said to be one of the 19th century’s greatest poets. At first his free-form style raised eyebrows, but it subsequently distinguished him as a stylistic innovator. He influenced the physician poet William Carlos Williams (of the Imagist movement) and Allen Ginsberg (of the Beat generation), among others.

Whitman was 42 years old, living in New York City, at the beginning of the Civil War. Among his seven siblings, his brother George was wounded in the Battle of Fredericksburg. When Whitman traveled to Virginia in search of George, he saw firsthand the casualties in the hospitals. His compassion moved him to make regular visits to help and console the injured. Though sometimes described as a “nurse,” he was not trained in that profession, which was evolving. Florence Nightingale in the Crimean War (1854-1856) and Clara Barton in the American Civil War (1861-1865) are noted for influencing the concept of a nursing profession capitalizing on women in a time when nursing of any sort was dominated by men.

Whitman, however, looked at himself more as a visitor and consolatory rather than a nurse. Nonetheless, he dutifully dressed wounds, assisted with amputations, and administered medications while consoling the wounded. Many lines of Stanza 3, in particular, describe his rounds: “I undo the clotted lint, remove the slough, wash off the matter and blood,” “I dress in the side, deep, deep, deep,” “I dress the perforated shoulder, the foot with the bullet-wound,” and “Cleanse the one with a gnawing and putrid gangrene, so sickening, so offensive.” His hospital rounds codified experiences and emotions that accounted for many Civil War writings. “The Wound Dresser” was not the only poem Whitman wrote about the Civil War and his hospital rounds. *Drum-Taps* and the *Sequel to Drum-Taps* (both 1885) contained reflections on the Civil War, as do two well-known poems written in response to Abraham Lincoln’s murder: “O Captain! My Captain!” and “When Lilacs Last in the Dooryard Bloom’d.”

In 1873, Whitman suffered a stroke and moved in with his brother, who resided in Camden, New Jersey, but there was little to be done for this malady at that time. “The Wound Dresser,” as well as others in *Drum-Taps*, are deeply moving and inspirational poems about Whitman’s role as a healthcare provider of sorts and remind us of the dimensions of the profession. Though the poems are often horribly graphic and serve to remind us of the evils of war, they also show sensitivity and can prompt readers to remember humankind’s ability to be compassionate.

